# Supplementary material for: Prevalence and risk factors of underweight among under-5 children in Bangladesh: Evidence from a countrywide cross-sectional study
Source: PLoS One. 2023 Apr 24;18(4):e0284797. doi: 10.1371/journal.pone.0284797 (PMC10124832; doi:10.1371/journal.pone.0284797)
Supplement: Attachment — Submitted filename: Response to Reviewers.pdf [file pone.0284797.s002.pdf]

Author responses to the review comments:

We would like to express our sincere gratitude to the reviewers and the Editors for their valuable comments. We have considered all the comments made by the reviewers and thoroughly revised and formatted the manuscript accordingly. A detailed response to each of the comments is provided in below:

**Academic Editor comments:**	**Response**	**Note**
Please include the following items when submitting your revised manuscript:<br>A rebuttal letter that responds to each point raised by the academic editor and reviewer(s). You should upload this letter as a separate file labeled 'Response to Reviewers'.<br>A marked-up copy of your manuscript that highlights changes made to the original version. You should upload this as a separate file labeled 'Revised Manuscript with Track Changes'.<br>An unmarked version of your revised paper without tracked changes. You should upload this as a separate file labeled 'Manuscript'.<br>If you would like to make changes to your financial disclosure, please include your updated statement in your cover letter. Guidelines for resubmitting your figure files are available below the reviewer comments at the end of this letter.	Thank you very much for carefully checking the manuscript and providing insightful comments.<br>All required files are uploaded to the journal system.	Revised texts are in red color.
**Journal Requirements:**	**Response**	**Note**
When submitting your revision, we need you to address these additional requirements.<br>1. Please ensure that your manuscript meets PLOS ONE's style requirements, including those for file naming. The PLOS ONE style templates can be found at<br>https://journals.plos.org/plosone/s/file?id=wjVg/PLOSOne_formatting_sample_main_body.pdf and<br>https://journals.plos.org/plosone/s/file?id=ba62/PLOSOne_formatting_sample_title_authors_affiliations.pdf	Thanks. We revised the manuscript following the PLOS ONE style.	Revised texts are in red color.<br>Page: 1-16
2. Please provide additional details regarding participant consent. In the ethics statement in the Methods and online submission information, please ensure that you have specified (1) whether consent was informed and (2) what type you obtained (for instance, written or verbal, and if verbal, how it was documented and witnessed). If your study included minors, state whether you obtained consent from parents or guardians. If the need for consent was waived by	Thanks. We add it in the Methods section.	Revised texts are in red color.<br>Page: 5

the ethics committee, please include this information. If you are reporting a retrospective study of medical records or archived samples, please ensure that you have discussed whether all data were fully anonymized before you accessed them and/or whether the IRB or ethics committee waived the requirement for informed consent. If patients provided informed written consent to have data from their medical records used in research, please include this information.		
3. Your ethics statement should only appear in the Methods section of your manuscript. If your ethics statement is written in any section besides the Methods, please move it to the Methods section and delete it from any other section. Please ensure that your ethics statement is included in your manuscript, as the ethics statement entered into the online submission form will not be published alongside your manuscript.	Thanks. We add it in the Methods section.	Revised texts are in red color. Page: 5
**Reviewer 1 comments:**	**Response**	**Note**
1. Title of the manuscript is quite confusing. In the title authors mentioned undernutrition but the concept of undernutrition couldn't be found in the main text. The authors should clearly define the key concepts.	Thank you very much for carefully checking the manuscript and providing insightful comments. We revise the title of the manuscript.	Revised texts are in red color. Page: 1
2. The abstract section of the manuscript is good and precise which makes more clear to the readers.	Thanks for the inspiration.	
3. Introduction part needs revision. If authors could add more details in the introduction that would be more impactful for the readers. The clarity part seems missing which needs to be added by the revision of the same.	We appreciate your comments. We revise the Introduction section.	Revised texts are in red color. Page: 2-4
4. In the introduction part it is not clear why the authors only focused on the underweight. What types of undernutrition prevails? What is malnutrition? What is the difference between malnutrition and undernutrition? However, it would be more relevant if the authors could add some more relevant studies which can highlight the gaps in the existing literature. Why and how sequential quantile regression is relevant to minimize the gaps?	Thanks. You know, undernutrition includes wasting, stunting, being underweight, and inadequate vitamins or minerals. On the other hand, malnutrition includes stunting, wasting, and underweight. We appreciate your concern. Basically, this study focused only on the underweight. Thus, we revise the title of the manuscript. We also revise the manuscript as per your comments.	Revised texts are in red color. Page: 3-4

5. The detailed data and estimation method section seem fine and authors appreciation for the same.	We are grateful to you for this kind of appreciation. It motivates us to continue research.	
6. On page number 14, it would be better to start as mothers rather than 'moms had a secondary or higher education….'. Mom's word does not sound well.	We are thankful to you for carefully checking the manuscript. We agreed with you and it is revised.	Revised texts are in red color.<br>Page: 14
7. The conclusion section needs refinement. The paper needs to jump swiftly into the conclusion by explaining why all the results are notable and important. However, the authors do not explain the policy implications properly. If authors could do that it would be better.	Thanks for highlighting this point. We revise the Conclusion section.	Revised texts are in red color.<br>Page: 15
8. Results and discussion section seems fine and would be appreciated.	We are thankful to you for your appreciative comments and feedback.	
9. However, I would like to insist that the authors should explain a bit that what are the study limitation.	Thanks. We add the strength and limitations section in the revised version.	Revised texts are in red color.<br>Page: 14-15
**Reviewer 2 comments:**	**Response**	**Note**
The topic is very relevant in the context of the increasing undernutrition among children in LMIC, particularly in Bangladesh. However, it needs a few modifications as discussed below.<br>1. In the abstract, it is necessary to mention the reason for using the quantile regression models.	Thanks. We appreciate your comments. We have revised the Abstract section as per your feedback.	Revised texts are in red color.<br>Page: 1
2. In the Introduction section, it is recommended to update the recent statistics about undernutrition among under-5 children in the global and Bangladesh context.	Thank you very much. This section is revised as per the comment.	Revised texts are in red color.<br>Page: 2
3. In the Methodology section, the authors mentioned "The variables included in this study were chosen based on their availability in the dataset, self-efficacy, as well as related existing research". It is strongly recommended to add citations for "existing research".	Thanks for pointing out this. We add references as per your suggestion.	Revised texts are in red color.<br>Page: 5
4. As per PloS ONE guideline, "Ethical statements" should be placed in the Methods section	Thanks. We move the Ethical statement in the Methods section following the PloS ONE style.	Revised texts are in red color.<br>Page: 5
5. The Result and Discussion are well written.	Thank you very much for your appreciative comments.	
6. Add strengths and limitations of this study.	Thanks. We add the strength and limitations section in the revised version.	Revised texts are in red color.<br>Page: 14-15

7. The manuscript needs thorough typos and language correction.	Thanks. We check and correct all typos and grammatical mistakes.	Revised texts are in red color.<br>Page: 1-15
8. Check all the references in the reference list carefully and delete unnecessary information for example “[Internet]”, “[cited 2022 Jan 8]”, and so on. Moreover, use “Available from: …” only for online documents.	Thank you very much for your careful checking of the manuscript. We use Mendeley for citation and follow the PLOS ONE style for correcting the references.	
9- The English should be revised	Thanks. We check and correct all typos and grammatical mistakes.	Revised texts are in red color.<br>Page: 1-15
10- A future work section should be added	Thank you very much. We add it to the revised manuscript.	Revised texts are in red color.<br>Page: 15

Finally, the revised manuscript has been produced following the valuable comments and suggestions of the reviewers. Once again, we would like to thank the reviewers for their sincere dedication, professional insights, and earnest cooperation in reviewing the manuscript.
