# Supplementary material for: Prevalence and risk factors of underweight among under-5 children in Bangladesh: Evidence from a countrywide cross-sectional study
Source: PLoS One. 2023 Apr 24;18(4):e0284797. doi: 10.1371/journal.pone.0284797 (PMC10124832; doi:10.1371/journal.pone.0284797)
Supplement: Attachment — Submitted filename: PONE-D-23-00547review.docx [file pone.0284797.s001.docx]

**Prevalence and risk factors of undernutrition among under-5 children in Bangladesh: evidence from a countrywide cross-sectional study (PONE-D-23-00547)**

This manuscript seems good. Minor revision needed. Author should have revised the manuscript.

1. Title of the manuscript is quite confusing. In the title authors mentioned undernutrition but the concept of undernutrition couldn’t be found in the main text. The authors should clearly define the key concepts.
2. The abstract section of the manuscript is good and precise which makes more clear to the readers.
3. Introduction part needs revision. If authors could add more details in the introduction that would be more impactful for the readers. The clarity part seems missing which needs to be added by the revision of the same.
4. In the introduction part it is not clear why the authors only focused on the underweight. What types of undernutrition prevails? What is malnutrition? What is the difference between malnutrition and undernutrition? However, it would be more relevant if the authors could add some more relevant studies which can highlight the gaps in the existing literature. Why and how sequential quantile regression is relevant to minimize the gaps?
5. The detailed data and estimation method section seem fine and authors appreciation for the same.
6. On page number 14, it would be better to start as mothers rather than ‘moms had a secondary or higher education….’. Mom’s word does not sound well.
7. The conclusion section needs refinement. The paper needs to jump swiftly into the conclusion by explaining why all the results are notable and important. However, the authors do not explain the policy implications properly. If authors could do that it would be better.
8. Results and discussion section seems fine and would be appreciated.
9. However, I would like to insist that the authors should explain a bit that what are the study limitation.
